# Visco- and poroelastic contributions of the zona pellucida to the mechanical response of oocytes

**DOI:** 10.1007/s10237-020-01414-4

**Published:** 2021-02-03

**Authors:** Alberto Stracuzzi, Johannes Dittmann, Markus Böl, Alexander E. Ehret

**Affiliations:** 1 Institute for Mechanical Systems, ETH Zurich, 8092 Zurich, Switzerland; 2 Empa, Swiss Federal Laboratories for Materials Science and Technology, 8600 Dübendorf, Switzerland; 3 Institute of Mechanics and Adaptronics, Technische Universität Braunschweig, Braunschweig, 38106 Germany

**Keywords:** Oocyte, Compression, Indentation, Anisotropy, Poro-viscoelasticity, Finite element simulation

## Abstract

Probing mechanical properties of cells has been identified as a means to infer information on their current state, e.g. with respect to diseases or differentiation. Oocytes have gained particular interest, since mechanical parameters are considered potential indicators of the success of in vitro fertilisation procedures. Established tests provide the structural response of the oocyte resulting from the material properties of the cell’s components and their disposition. Based on dedicated experiments and numerical simulations, we here provide novel insights on the origin of this response. In particular, polarised light microscopy is used to characterise the anisotropy of the zona pellucida, the outermost layer of the oocyte composed of glycoproteins. This information is combined with data on volumetric changes and the force measured in relaxation/cyclic, compression/indentation experiments to calibrate a multi-phasic hyper-viscoelastic model through inverse finite element analysis. These simulations capture the oocyte’s overall force response, the distinct volume changes observed in the zona pellucida, and the structural alterations interpreted as a realignment of the glycoproteins with applied load. The analysis reveals the presence of two distinct timescales, roughly separated by three orders of magnitude, and associated with a rapid outflow of fluid across the external boundaries and a long-term, progressive relaxation of the glycoproteins, respectively. The new results allow breaking the overall response down into the contributions from fluid transport and the mechanical properties of the zona pellucida and ooplasm. In addition to the gain in fundamental knowledge, the outcome of this study may therefore serve an improved interpretation of the data obtained with current methods for mechanical oocyte characterisation.

## Introduction

In the recent decades, mechanical properties have been recognised as important indicators of cell state, e.g. related to diseases (Suresh 2007), differentiation (Plusa and Hadjantonakis 2016), or drug screening and evaluation (Krishnan et al. 2016). Prospective applications include the identification of cancer cells through AFM testing (Suresh 2007), the recognition of altered red blood cells with microfluidic devices (Rosenbluth et al. 2008), and the selection of oocytes for in vitro fertilisation (IVF) procedures (Yanez et al. 2016).

**Fig. 1:**
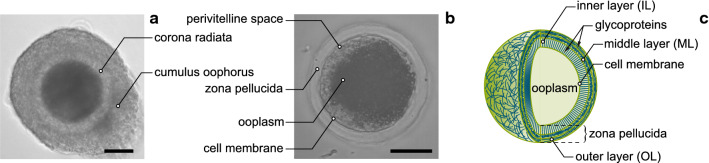
Brightfield image of a cumulus–oocyte complex consisting of the oocyte, the corona radiata, and several cumulus cell layers (**a**). Brightfield image of OP and ZP, clearly recognisable as a thick layer, after removal of the cumulus cells and the corona radiata (**b**). Idealised illustration of the oocyte (**c**). While the glycoproteins of the inner and outer layer have a clear orientation, no distinct alignment of the proteins could be detected for the middle layer. Scale bars: $50\;\upmu \hbox {m}$

Commonly, the experimental measurements are quantified by very few parameters that reflect the lumped response of the cell to the mechanical load, applied at its boundary. However, these responses are typically the result of an intricate combination of structural and material properties of the cell, activated through the external load. An improved understanding of how these global responses are generated can therefore strongly help interpreting the test data and enable to associate the measured quantities with physical and structural properties of the cell. Such knowledge can be obtained from dedicated experiments, theoretical models, and computer simulations. In the present work, we illustrate this for the mammalian oocyte, the female germ cell, whose mechanical properties have increasingly been studied during the last years. As noted above, this interest is mainly associated with attempts to assess the potential success of IVF based on the mechanical properties of the oocyte ( Liu et al. 2012; Yanez et al. 2016) as an alternative to morphological criteria (Leibfried and First 1979), whose efficacy is controversial in view of the difficulty to distinguish between developmental dysfunction (Meriano et al. 2001) and natural variability (Balaban and Urman 2006).

Compared to most other cells, oocytes have a distinguished structure (Fig. 1) that also affects their mechanical behaviour. Furthermore, it is well known that both the morphology (e.g. Cran 1985; Yi et al. 2013) and mechanical properties (Murayama et al. 2006; Andolfi et al. 2016) of the oocyte change with maturation. The ooplasm (OP) is surrounded by the oolemma, the perivitelline space, and the zona pellucida (ZP). Inside the cell, microtubules form a coarse, mesh-like network embedding the nucleus, and close to the oolemma an actin cortex is developed (Suzuki et al. 2003; Albertini 2015). The ZP is probably the most distinct feature when compared to other cells. Morphologically, it has a relatively large thickness (e.g. in pigs it is about $15\,\upmu \hbox {m}$ (Sinowatz et al. 2001), i.e. more than 15% of the typical radius of the porcine oocyte), and it is characterised by a porous network of glycoproteins (GPs) of varying compactness and alignment, often described as three-layered (Keefe et al. 1997; Pelletier et al. 2004; Shen et al. 2005; Raju et al. 2007; Familiari et al. 1992, 2006; Fléchon et al. 2004; Michelmann et al. 2007; Novo et al. 2012). The ZP not only regulates the interactions between oocyte and sperm, but also bears the major part of the loads acting on the cell (e.g. Abadie et al. 2014; Shen et al. 2019), in contrast to most other cells, for which the actin cortex is mainly responsible for carrying the mechanical loads (Salbreux et al. 2012).

Biomechanical works studied the mechanical properties of the whole oocytes by means of compression experiments (Cole 1932; Hiramoto 1963, 1976; Sakuma et al. 2013; Abadie et al. 2014; Gana et al. 2017; Dittmann et al. 2018; Shen et al. 2019; Andolfi et al. 2019), sessile drop experiments (Hiramoto and Yoneda 1986), microinjection, beam deflection or indentation methods (Hiramoto 1976; Sun et al. 2003; Kim et al. 2004; Wacogne et al. 2008; Huang et al. 2009; Liu et al. 2010, 2012; Khalilian et al. 2013; Abadie et al. 2014; Dittmann et al. 2018; Shen et al. 2019), tactile measurement methods (Murayama et al. 2004), magnetic bead/particle (tracking) methods (Hiramoto 1969), classical micropipette aspiration (Shôji et al. 1978; Drobnis et al. 1988; Khalilian et al. 2010; Zhao et al. 2013; Yanez et al. 2016), and cell elastimetry techniques (Mitchison and Swann 1954; Nakamura and Hiramoto 1978). Further works analysed the mechanical properties of the ZP with atomic force microscopy (Papi et al. 2009, 2013; Boccaccio et al. 2012, 2014; Andolfi et al. 2016), indentation (Khalilian et al. 2013), micropipette aspiration (Drobnis et al. 1988; Khalilian et al. 2010), and elastimetry (Mitchison and Swann 1954; Hiramoto 1963).

These experimental studies were often accompanied by simple mathematical models to obtain estimates of a lumped elastic stiffness of the oocyte. In this way, based on the assumption of linear elastic material behaviour, point-load (Sun et al. 2003) and micropipette aspiration (Khalilian et al. 2010; Zhao et al. 2013) models were established, and the modified Hertz theory was used to interpret nanoindentation tests (e.g. Papi et al. 2009). Only few modelling attempts have made use of the information on cell geometry and structure, and proposed 2D or 3D finite element (FE) models of the oocyte, the OP, or ZP (Liu et al. 2012; Boccaccio et al. 2012, 2014; Shen et al. 2019). In these models, however, neither the anisotropic properties of the ZP nor potential solid–liquid interactions of the moving cellular fluid within the porous protein network in the ZP were considered.

Previous work revealed that the ZP undergoes large changes of volume when the oocyte is subjected to slow, quasi-static compression and indentation tests (Dittmann et al. 2018). Given the high bulk modulus of water, the observed compressibility points at liquid transport over the cell boundaries, and, in general, this flow suggests time-dependent behaviour due to the finite permeability experienced by the fluid. In fact, several works evidence that the oocyte as a whole (Hiramoto 1976; Liu et al. 2012; Sakuma et al. 2013; Yanez et al. 2016; Shen et al. 2019), but also the ZP (Nakamura and Hiramoto 1978; Kim 2013; Papi et al. 2013; Boccaccio et al. 2014) and the OP (Shôji et al. 1978; Hiramoto 1969) alone display time-dependent characteristics. However, such time-dependent effects were so far associated with viscoelasticity and considered in terms of lumped rheological models (Kim 2013; Yanez et al. 2016) connecting the measured time-varying force or pressure with typically one-dimensional measures of displacements or strains. More advanced models were proposed based on hyper-viscoelasticity (Boccaccio et al. 2014) or dynamic network theory (Shen et al. 2019) but the oocyte and its components were considered as incompressible and monophasic materials, neglecting any potential relative motion between solid and liquid phases.

In the present work, we present novel data on the time-dependent behaviour of oocytes. In particular, both their overall force response and the deforming geometry are recorded, allowing the quantification of volume changes of the ZP and OP in compression and indentation. These data are supplemented by microstructural information, namely birefringence data associated with the realignment of GPs within the ZP. In order to rationalise the observed behaviours, we use and adapt a biphasic anisotropic hyper-viscoelastic constitutive model (Ehret et al. 2017; Wahlsten et al. 2019), which interprets the overall compressibility of the ZP as a loss of liquid across the outer boundary. Specifically, plate–plate relaxation compression experiments were used to calibrate the model parameters, whereas plate–plate cyclic tests as well as force, volume, and deformation in indentation tests were acquired to validate the predictive capability of the implemented model. Indentation tests generate a substantially different and more localised state of deformation, which is akin to the state experienced by oocytes in IVF techniques. For the first time, the observed time-dependent behaviour is thus rationalised in terms of both visco- and poroelastic contributions, and the timescales associated with these processes are identified. The proposed model describes with good accuracy the macroscopic response of the oocyte, shows favourable predictive qualities, and sheds light on the microstructural changes in the ZP upon compressive deformations. The present contribution is not concerned with the distinction between oocytes in different states of health or maturation but aims at explaining the mechanical response in single-cell compressive tests in terms of physical properties and structure of the oocyte. To this end, porcine oocytes are used for this fundamental research on mammalian egg cells, which contributes to the basic understanding of the mechanical behaviour of this special cell type. Nevertheless, the general findings of this study may serve an improved interpretation of the data obtained with the methods that are currently used for the mechanical characterisation of oocytes.

## Materials and experimental methods

### Oocyte collection and preparation

In the present study, immature oocytes in the germinal vesicle stage were considered. Ovaries from three-month-old pigs (*Sus scrofa domestica*) were obtained from a slaughterhouse immediately after animal killing and stored in physiological saline solution at $34\,^{\circ }\hbox {C}$ to $39\,{^{\circ }}\hbox {C}$. The cumulus–oocyte complexes were flushed out and denudated using denudation pipette with an inner diameter of $150\,\upmu \hbox {m}$ and denudation solution ($9.65\hbox { g l}^{-1}$ Dulbecco’s phosphate-buffered saline, $0.1\hbox { g l}^{-1}$ $\hbox {CaCl}_{2}$, $3\hbox { g l}^{-1}$ bovine serum albumin (BSA), $50\hbox { mg l}^{-1}$ gentamicin). Before realising the experiments, oocytes were kept in culture medium ($10.6\hbox { g l}^{-1}$ TCM199 (M0393, Sigma-Aldrich), $0.35\hbox { g l}^{-1}$ sodium bicarbonate, $22\hbox { mg l}^{-1}$ sodium pyruvate, $100\hbox { mg l}^{-1}$ BSA, $50\hbox { mg l}^{-1}$ gentamicin) at $39\,{^{\circ }}\hbox {C}$. The culture medium was covered with mineral oil (M8410, Sigma-Aldrich) to prevent evaporation and improve the temperature stability during the experiments.

**Fig. 2:**
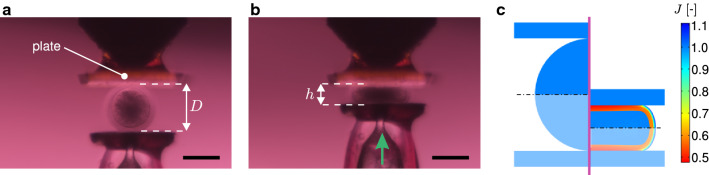
Compression load case: **a** Microscope image of the experimental set-up, **b** deformed configuration at $\delta = 0.6$, and **c** 2D field of the local volume ratio *J* calculated by the FE model, in both reference (left) and deformed (right) configurations (the non-shaded area represents the actual domain adopted for simulations due to symmetry). Scale bars: $100\,\upmu \hbox {m}$

**Fig. 3:**
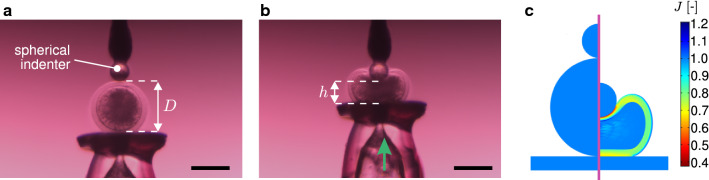
Indentation load case: **a** microscope image of the experimental set-up, **b** deformed configuration at $\delta = 0.6$, and **c** 2D field of the local volume ratio *J* calculated by the FE model, in both reference (left) and deformed (right) configurations. Scale bars: $100\,\upmu \hbox {m}$

### Micromechanical experiments

Micromechanical experiments were realised with a custom-made set-up embedded in an inverted microscope (Nikon Ti Eclipse) (cf. Dittmann et al. 2018). All experiments were performed in a basin, filled with culture medium, and positioned on a heated microscope stage [HT 300, Minitube GmbH, temperature during sampling ($38.5 \pm 1.0)\,{^{\circ }}\hbox {C}$]. During the tests, oocytes were fixed by a suction micropipette (MPH-XLG-30, Origio), glued to a piezo-micromanipulator (FT-RS1002 Microrobotic System, FemtoTools, Switzerland), and moved against a plate, for compression, or a sphere (diameter $50\,\upmu \hbox {m}$), for indentation, both glued to the tip of a force sensor (FT-S1000, FemtoTools, Switzerland). The entire set-up exhibits an overall stiffness of approximately $180\,\upmu \hbox {N}\, \upmu \hbox {m}^{-1}$, i.e. significantly higher than the expected values when sampling the cells.

For all tests, the maximum deformation state was defined by $\delta =u/D=(D-h)/D=0.6$ (Figs. 2a, b, 3a, b), where *u* and *D* are the applied displacement and the undeformed external diameter of the oocyte, respectively.

Compression ($n=7$) and indentation ($n=7$) relaxation experiments were realised by first rapidly compressing the oocyte to $\delta =0.6$ at a mean rate of $5.25\, \upmu \hbox {m s}^{-1}$ and then holding the deformation for 300 s. Corresponding single cycle quasi-static experiments at a loading velocity of $0.2\,\upmu \hbox {m s}^{-1}$ had been performed before (Dittmann et al. 2018), whose hysteresis curves are considered here. Reaction forces were recorded with a frequency of $10\hbox { Hz}$.

### Quantification of volumes

The volumes of the ZP and the OP were determined from the recorded images of the median plane of the oocytes, assuming axisymmetry (cf. Dittmann et al. 2018). ZP and OP volumes were approximated by summing the volumes of the tori obtained by rotating the respective square-shaped pixel area $a_p$ about the vertical centre line. The corresponding cubature formula reads

1 $$\begin{aligned} V_{\alpha } \approx \pi a_p \sum _{k=1}^{N_{\alpha }} r_k, \end{aligned}$$

where $\alpha =\{ \mathrm {OP},\mathrm {ZP}\}$ indicates the considered domain, $N_{\alpha }$ is the total number of pixels in the domain $\alpha$, and $r_k$ is the distance of the centre of the *k*th pixel from the vertical centre line. We also note that the perivitelline space has been assigned to the OP, as oocytes feature a plasma membrane that is partially detached from the ZP (Fig. 1b).

For the relaxation experiments, during the loading path ZP and OP individual areas were analysed at four equidistantly distributed time points (i.e. $\delta =0/0.2/0.4/0.6$), whereas during the relaxation phase the images at 150 s and 300 s after the loading were considered. For the acquisition and processing of the images, custom-made Matlab scripts ($\hbox {Matlab}^{\textregistered }$ R2016b, The MathWorks, Inc.) were used.

### Determination of the optical anisotropy in ZP

The OpenPolScope technology (Oldenbourg et al. 1998), based on polarised light microscopy, was utilised to determine the anisotropy of the ZP, by measuring the slow axis orientation $\theta _{\mathrm{PS}}$, usually aligned with the local anisotropy, and the retardance $r_{\mathrm{PS}}$, i.e. the product of sample thickness and optical anisotropy. The latter is defined by the difference of the refractive index for two perpendicular polarised beams. The retardance represents a measure of how strong the incoming light will change its polarisation state. Consequently, when $r_{\mathrm{{PS}}}=0$, no optical anisotropy in terms of polarisation can be assumed.

To compute $r_{\mathrm{{PS}}}$ and $\theta _{\mathrm{{PS}}}$, the system was equipped with a liquid crystal device to electronically manipulate the polarisation state and to automatically record images of five polarisation states. A background correction (Shribak and Oldenbourg 2003) was applied to limit light path irregularities. A previously configured Köhler illumination (air condenser, LWD 0.52 Condenser, 30 mm WD Nikon) was adopted to ensure optimal illumination conditions.

Undeformed ($n=21$) and compressed ($n=7$) oocytes were imaged, and linescans, i.e. readouts of $\theta _{\mathrm{{PS}}}$ and $r_{\mathrm{{PS}}}$ along a straight line, respectively, were performed across the ZP at two different locations. To represent all the data together on the interval [0,1], they were plotted against the normalised thickness ${\hat{t}}$ or ${\hat{t}}'$, referring to the undeformed or deformed state, respectively. To this end, the distance information of each line scan was divided by the local thickness of the ZP, i.e. the length of the line. Moreover, for the sake of improved comparability, the orientation data were shifted such that the average of the angles coincides with $\theta =0{^{\circ }}$ at the inner radius in the undeformed state and with $\theta =90{^{\circ }}$ at the outer radius in the deformed state.

## A poro-viscoelastic model of the oocyte

A continuum model of the oocyte has been implemented, which includes a poro-viscoelastic description of the ZP and a hyperelastic (nearly incompressible) OP. In this framework, ${\varvec{\chi }}({\varvec{X}},t)$ is the mapping which describes the motion of a material particle from the reference position ${\varvec{X}}$ to the current one ${\varvec{x}}={\varvec{\chi }}({\varvec{X}},t)$. The local deformation is described by the tangent map ${\mathbf {F}}={\mathrm{Grad}}{{\varvec{\chi }}}({{\varvec{X}}},t)$, $J=\mathrm {det}{\mathbf {F}}>0$ is the local volume ratio, and the right Cauchy–Green tensor is defined as ${\mathbf {C}}={\mathbf {F}}^{\mathrm {T}}{\mathbf {F}}$ (see e.g. Marsden and Hughes 1994).

### Zona pellucida

Based on the chemoelastic approach presented in Stracuzzi et al. (2018), the ZP is modelled as a saturated biphasic material, consisting of incompressible solid and fluid constituents. The quantities $\phi _{\mathrm s} = \phi _{\mathrm s}^{\mathrm {ref}}/J$ and $\phi _{\ell }$ define the solid and liquid spatial volume fractions, respectively, where $\phi _{\mathrm s}^{\mathrm {ref}}$ is the solid volume fraction in the reference state. The set of governing equations is given by the balances of mass and linear momentum of the material, assuming that body forces and inertial terms are negligible (e.g. Ehlers et al. 2008; Stracuzzi et al. 2018), respectively,

2 $$\begin{aligned} {\mathrm{div}}{\varvec{\sigma }}={\varvec{0}}, \qquad {\dot{J}}=J {\mathrm{div}}{\varvec{q}}. \end{aligned}$$

**Fig. 4:**
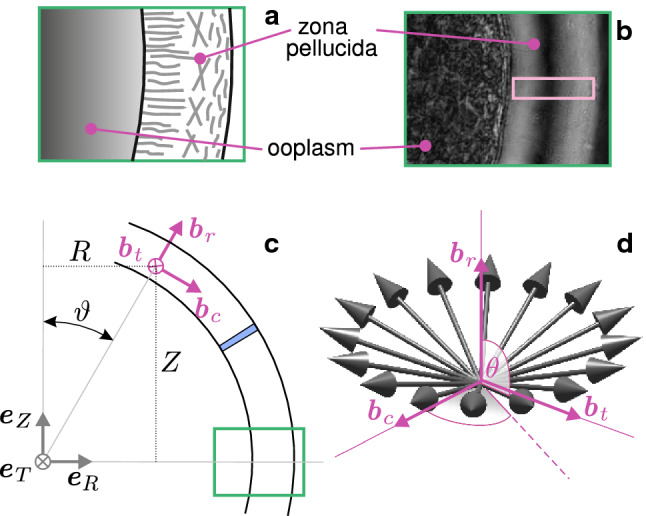
Qualitative sketch representing modelled oriented structures within ZP (**a**) and PolScope image of the ZP (**b**) with the detail of the region analysed using PolScope imaging (purple rectangle). Global and local reference systems (**c**) and 3D set of fibres ($N=16$, for representation reasons) used to describe ZP anisotropy (**d**)

The tensor $\varvec{\sigma }$ and the vector ${\varvec{q}}$ represent the Cauchy stress tensor and the fluid flux vector, respectively. The latter is constitutively prescribed according to a Darcy-type law and reads (Ehlers et al. 2008; Stracuzzi et al. 2018)

3 $$\begin{aligned} {\varvec{q}}=-{\mathbf {k}} {\mathrm{grad}}{\mu }, \end{aligned}$$

where ${\mathbf {k}}$ and $\mu$ are the spatial hydraulic conductivity tensor and the fluid chemical potential, respectively. From thermodynamic considerations, the Cauchy stress tensor can be shown to take the general form (Stracuzzi et al. 2018)

4 $$\begin{aligned} \varvec{\sigma } = \varvec{\sigma }_{\mathrm m} - \mu {\mathbf {I}}, \qquad \varvec{\sigma }_{\mathrm m}=\frac{2}{J} {\mathbf {F}}\frac{\partial \varPsi _{\mathrm m}}{\partial {\mathbf {C}}}{\mathbf {F}}^{\mathrm {T}}, \end{aligned}$$

where $\varPsi _{\mathrm m}$ defines the strain energy density function of the solid constituent and ${\mathbf {I}}$ denotes the identity tensor. To include the information from the PolScope measurements (Fig. 4a), the anisotropic distribution of the GPs (Fig. 4b) is modelled by means of a discrete set of representative fibre families ${\varvec{M}}^i$, $i = 1,2,\ldots N$ (with *N* an even integer), which are defined in the reference configuration (Fig. 4c, d) and which deform affinely according to the transformation ${\varvec{m}}^i= \mathbf {F} {\varvec{M}}^i$ (Mauri et al. 2015b). We remark that the use of a few representative fibre families to capture effects caused by the presence of a much greater number of physical fibres or filaments is a common strategy in biomechanics of collagenous tissues (see e.g. Holzapfel 2000; Buerzle and Mazza 2013), that was adopted here. However, the properties of such a single fibre cannot directly be equated with those of a GP filament but as the ensemble response of many aligned filaments.

The unit vectors ${\varvec{M}}^i$ are equiangularly distributed so that quasi-transverse isotropy with respect to the radial direction ${\varvec{b}}_r$ is obtained (Fig. 4d). The *i*th material unit vector in terms of the local spherical coordinate system $\{ {\varvec{b}}_r(R,Z),{\varvec{b}}_t(R,Z),{\varvec{b}}_c(R,Z) \}$ is then given by (cf. Mauri et al. 2015a; Wahlsten et al. 2019)

5a $$\begin{aligned} {\varvec{M}}^i&= {\mathrm {sin}}\theta {\mathrm {cos}}\varphi ^i {\varvec{b}}_c+ \mathrm {sin}\theta \mathrm {sin}\varphi ^i {\varvec{b}}_t+ \mathrm {cos}\theta {\varvec{b}}_r , \end{aligned}$$

5b $$\begin{aligned} \varphi ^i&=\frac{2\pi }{N}\left( i-\frac{1}{2} \right) , \quad i=1,2,\ldots ,N. \end{aligned}$$

**Fig. 5:**
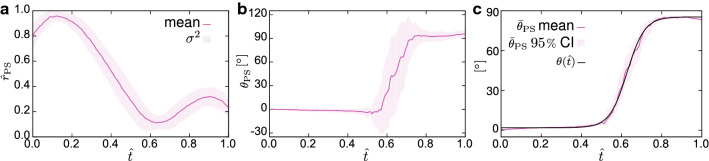
Normalised retardance ${\hat{r}}_{\mathrm{{PS}}}$ (**a**) and orientation $\theta _{\mathrm{{PS}}}$ of the slow axis (**b**) versus the normalised thickness ${\hat{t}}$ of the ZP in the undeformed state. Fitted analytical curve representing the orientation in ZP (**c**) after the post-processing described in Sect. 3.1. Parameter values: $a=-14.36$, $b=22.87$ , $\gamma '=4.641{^{\circ }}$, $\gamma '' = 88.12{^{\circ }}$. Moreover, the shaded area in **c** represents the 95% confidence interval (CI) for the mean, calculated with BCA bootstrap and $10\times 10^-5$ resamples. In all the figures, ${\hat{t}}=0$ and ${\hat{t}}=1$ correspond to internal and external radii, respectively

Their inclination angle $\theta =\theta (R,Z)$ with respect to ${\varvec{b}}_r$ is assigned consistently with the measured orientation (Fig. 5). In particular, since the orientations $\theta$, $-\theta$, $(180{^{\circ }}+ \theta )$, and $(180{^{\circ }}-\theta )$ are all represented by the same discrete set of fibres (cf. Eq. 5), the measured orientations $\theta _{\mathrm{{PS}}}$ were accordingly mapped onto the interval $[0,90]{^{\circ }}$, and the converted data ${\bar{\theta }}_{\mathrm{{PS}}}$ (Fig. 5c) was fitted by the continuous empiric relation

6 $$\begin{aligned} \theta ({\hat{t}})=90{^{\circ }}-\gamma ' + \frac{\gamma ' - \gamma ''}{1+\mathrm {e}^{(a+b{\hat{t}})}}, \end{aligned}$$

written as a function of the normalised thickness ${\hat{t}}$ and shown in Fig. 5c.

Apart from the viscous flow of the contained fluid, the viscoelastic behaviour of GPs is included as a second dissipative mechanism. This is described by defining each representative family of GPs by a viscoelastic element (cf. Nguyen et al. 2007; Wahlsten et al. 2019) consisting of an *equilibrium* part, whose stored elastic energy depends on the stretch $\lambda ^i_{\mathrm f}=||{\varvec{m}}^i ||=||{\mathbf {F}}{\varvec{M}}^i ||$, and a *non-equilibrium* part, whose stored elastic energy depends on the stretch $\lambda ^i_{\mathrm {fd}}=||{\varvec{m}}^i_{\mathrm {fd}} ||$. The time evolution of the internal variables ${\varvec{m}}^i_{\mathrm {fd}}$ is defined by (cf. Rubin 1994; Mauri et al. 2015a; Wahlsten et al. 2019)

7 $$\begin{aligned} \dot{{\varvec{m}}}^i_{\mathrm {fd}}={\mathbf {l}} {\varvec{m}}^i_{\mathrm {fd}}-\varGamma ^i {\varvec{m}}^i_{\mathrm {fd}}, \quad i=1,2,\ldots ,N, \end{aligned}$$

where ${\mathbf {l}}=\dot{{\mathbf {F}}}{\mathbf {F}}^{^{-1}}$ and with the initial condition ${{\varvec{m}}}^i_{\mathrm {fd}} (t=t_0) = {\varvec{M}}^i$. In Eq. (7), the scalars $\varGamma ^i$ represent the rate of fibre inelastic deformation (Rubin and Bodner 2002), constitutively specified in the next section.

#### Specific constitutive equations

The strain energy density of the ZP is adapted from the exponential models presented in Rubin and Bodner (2002), Mauri et al. (2015a), and Stracuzzi and Ehret (2016) and is defined by

8a $$\begin{aligned}&\varPsi _{\mathrm m} \left( {\mathbf {C}},\{\lambda ^i_{\mathrm f}\}_{i=1,2,\ldots ,N},\{ \lambda ^i_{\mathrm {fd}}\}_{i=1,2,\ldots ,N} \right) \nonumber \\&\quad =\phi _{\mathrm s}^{\mathrm {ref}} \left[ \frac{c_0}{2q}\left( \mathrm {e}^{qg}-1 \right) + U(J)\right] , \end{aligned}$$

8b $$\begin{aligned}&g = g_{\mathrm s}+g_{\mathrm f}+g_{\mathrm {fd}}, \end{aligned}$$

where $c_0$ and *q* are material parameters. *U*(*J*) is a penalisation term that is active only when $J \rightarrow \phi _{\mathrm s}^{\mathrm {ref}}$ and that was introduced for the sake of completeness to ensure the compaction limit (Federico and Grillo 2012). The exponent *g* in Eq. (8) is subdivided into an isotropic term linked to the matrix component of ZP and to the equilibrium and non-equilibrium GP contributions, respectively. These terms are specified by (cf. Wahlsten et al. 2019)

9a $$\begin{aligned}&g_{\mathrm s} (I_1,J) = {c_1} \left[ (I_1-3)+\frac{1}{c_2}(J^{-2c_2}-1) \right] , \end{aligned}$$

9b $$\begin{aligned}&g_{\mathrm f} \left( \{\lambda ^i_{\mathrm f}\}_{i=1,2,\ldots ,N}\right) = \frac{c_{\mathrm f}}{c_3}\frac{1}{N} \sum _{i=1}^N \left\langle \lambda _{\mathrm f}^i-1 \right\rangle ^{2c_3}, \end{aligned}$$

9c $$\begin{aligned}&g_{\mathrm {fd}} \left( \{ \lambda ^i_{\mathrm {fd}}\}_{i=1,2,\ldots ,N} \right) = \frac{c_{\mathrm {fd}}}{c_3}\frac{1}{N} \sum _{i=1}^N \left\langle \lambda _{\mathrm {fd}}^i-1 \right\rangle ^{2c_3}, \end{aligned}$$

where $I_1=\mathrm {tr}{{\mathbf {C}}}$, $\{ c_1,c_2,c_3,c_{\mathrm f},c_{\mathrm {fd}} \}$ are material parameters and $\left\langle \cdot \right\rangle$ denotes Macaulay brackets. Using Eq. (4)$_2$, the corresponding Cauchy stress contributions are obtained as

10 $$\begin{aligned} \varvec{\sigma }_{\mathrm m} = \frac{\phi _{\mathrm s}^{\mathrm {ref}}}{J}c_0\mathrm {e}^{qg} \left[ \varvec{\sigma }_{\mathrm s}+\frac{1}{N} \sum _{i=1}^N \left( \varvec{\sigma }_{\mathrm f}^{i} + \varvec{\sigma }_{\mathrm {fd}}^{i} \right) \right] , \end{aligned}$$

with

11a $$\begin{aligned}&\varvec{\sigma }_{\mathrm s} = {c_1} \left( {\mathbf {b}}-J^{-2c_2}{\mathbf {I}} \right) , \end{aligned}$$

11b $$\begin{aligned}&\varvec{\sigma }_{\mathrm f}^{i} = \frac{c_{\mathrm f}}{\lambda _{\mathrm f}^{i}} \left\langle \lambda _{\mathrm f}^i-1 \right\rangle ^{2c_3-1} {\varvec{m}}_{\mathrm f}^{i} \otimes {\varvec{m}}_{\mathrm f}^{i}, \end{aligned}$$

11c $$\begin{aligned}&\varvec{\sigma }_{\mathrm {fd}}^{i} = \frac{c_{\mathrm {fd}}}{\lambda _{\mathrm {fd}}^{i}} \left\langle \lambda _{\mathrm {fd}}^i-1 \right\rangle ^{2c_3-1} {\varvec{m}}_{\mathrm {fd}}^{i} \otimes {\varvec{m}}_{\mathrm {fd}}^{i}, \end{aligned}$$

where ${\mathbf {b}}={\mathbf {F}}{\mathbf {F}}^{\mathrm {T}}$ is the left Cauchy–Green tensor.

The viscoelastic behaviour of the GPs is defined through the rate of inelastic deformation, given by the phenomenological, nonlinear relation

12 $$\begin{aligned} { \varGamma ^i=\frac{1}{N\nu } \mathrm {e}^{-\beta \left( \lambda _{\mathrm f}^i/\lambda _{\mathrm {fd}}^i-1 \right) }[\phi _\mathrm {s}^{\mathrm {ref}} c_0 \mathrm {e}^{qg}\mathrm {tr}\varvec{\sigma }_{\mathrm {fd}}^i], \quad i=1,2,\ldots ,N, } \end{aligned}$$

with $\nu$ a viscosity-like material parameter, adapting the formulation presented in Mauri et al. (2015a).

The hydraulic conductivity is assumed to be spatially isotropic (Ateshian and Weiss 2010), so that

13 $$\begin{aligned} {\mathbf {k}}= k^{\mathrm{ZP}}(J) {\mathbf {I}}, \end{aligned}$$

with $k^{{\mathrm{Z}}\,P}(J)$ representing the *J*-dependent scalar hydraulic conductivity, here chosen in the form proposed in Holmes and Mow (1990), i.e.

14 $$\begin{aligned} k^{{\mathrm{ZP}}}(J)=k^{\mathrm {ref}}\left( \frac{J-\phi _\mathrm{s}^{\mathrm{ref}}}{1-\phi _{\mathrm{s}}^{\mathrm{ref}}} \right) ^{ \alpha _1} \mathrm {exp} \left[ \frac{1}{2}\alpha _2 \left( J^2 -1 \right) \right] , \end{aligned}$$

in order to represent an exponential decay of the conductivity value when the pores are compressed. In Eq. (14), $k^{\mathrm {ref}}$ is the reference scalar hydraulic conductivity and $\{ \alpha _1, \alpha _2\}$ are constant material parameters.

The problem defined through the equations in (2) is closed by assigning initial conditions for the displacement vector and the chemical potential and by prescribing boundary conditions to the linear momentum equation (2)$_1$ (either displacement or the stress) and to the mass balance (2)$_2$ (either the fluid flux or the chemical potential).

### Ooplasm

The OP mainly consists of a biorheological fluid (cytosol) (Shen et al. 2019), but shows also a structural behaviour in that it is capable of sustaining not only hydrostatic pressures but also transferring shear, as proved by the deformation of the nucleus under compression (see Sect. 4.2.2). Furthermore, even if the OP has been reported to exhibit time-dependent behaviour (see Shen et al. 2019), the estimated viscosity parameter associated with the OP is almost one order of magnitude lower than that of the ZP (Shen et al. 2019), thus resulting in shorter mechanical timescales. For this reason, we model the OP as an isotropic, hyperelastic material with nearly incompressible neo-Hookean strain energy density function (cf. Holzapfel 2000)

15 $$\begin{aligned} \varPsi ^{{\mathrm{OP}}}({\mathbf {C}}) = \frac{1}{2} c ( {\bar{I}}_1-3)+\frac{1}{2}\kappa (J-1)^2, \end{aligned}$$

built-in in the FE software (COMSOL 2015), where *c* and $\kappa$ are material parameters and ${\bar{I}}_1=\mathrm {tr}[J^{-\frac{2}{3}}{\mathbf {C}}]$.

### Finite element implementation

The model was implemented in the FE software COMSOL $\hbox {Multiphysics}^{\textregistered }$ (version 5.2, COMSOL AB, Stockholm, Sweden). Due to the assumed axisymmetry of the domain and loads (Figs. 2c, 3c), the modelled domains in the compression and indentation configurations were one quarter and one half of the whole oocyte section, respectively (Figs. 2c, 3c). The two loading configurations used in the experiments are replicated in the simulations. During testing, oocytes were immersed in cell culture medium (cf. Sect. 2.2). Hence, the natural Dirichlet boundary condition $\mu =0$ was applied to the outer surface of the ZP which is in contact with the external bath.

Moreover, where the loading plates and the spherical indenter get into contact with the ZP boundary, this Dirichlet boundary condition was suppressed and replaced by a no-flux condition at the respective nodes, thus influencing the fluid pathways within the ZP. Zero-flux conditions are applied also on the symmetry boundaries. Furthermore, since no net fluid exchange between ZP and OP is observed (cf. Sect. 4.2), in the following simulations no flux is allowed through the interface between ZP and OP, and continuity of the displacement field is assumed. The contact between the oocyte and the plates or the spherical indenter was modelled as frictionless and by means of a penalty method. In all the simulations, the number of representative fibre families was set to $N=32$, whereas *D* and the ZP thickness $t^{\mathrm{{ZP}}}$ were obtained from the average values of the geometry data of the oocytes tested in compression relaxation, i.e. $D=142.71 \pm 5.74 \,\upmu \hbox {m}$ and $t^{\mathrm{{ZP}}}=14.43 \pm 0.79 \, \upmu \hbox {m}$.

**Fig. 6:**
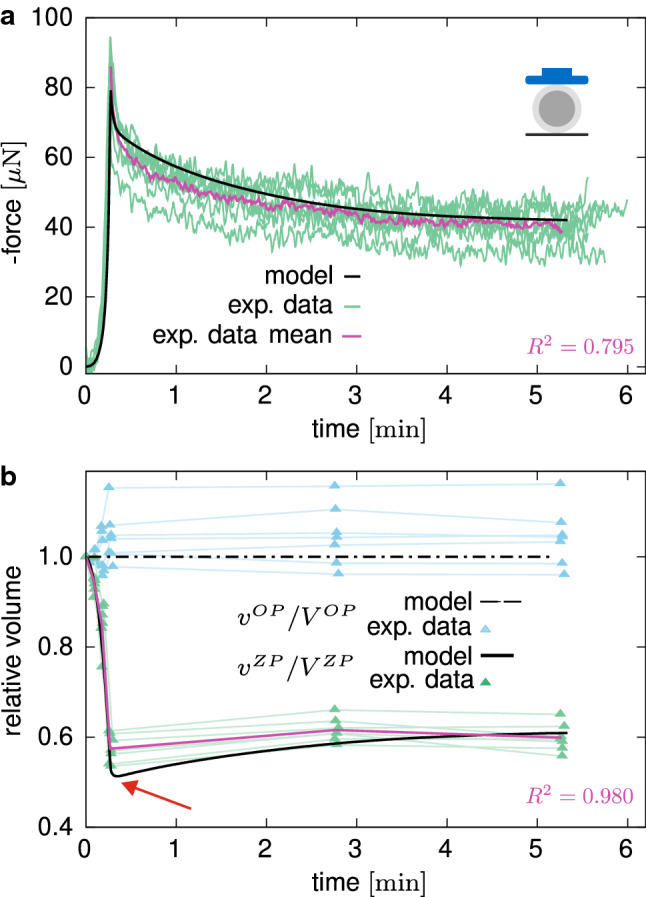
Compression by means of a flat plate: measured force in relaxation experiment (**a**) and relative volume change (**b**) versus time. The mean curves used for fitting are plotted in purple and are used for the evaluation of the coefficient of determination $R^2$, here taken as an approximate measure for the quality of the fit (Kvålseth 1983)

**Fig. 7:**
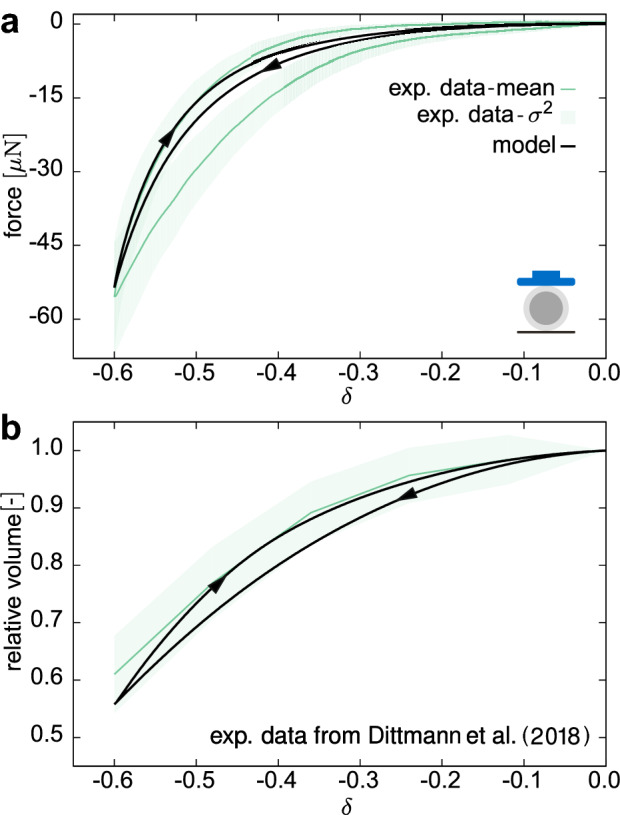
Compression by means of a flat plate: measured force during the first cycle of a slow rate applied displacement (**a**) and relative volume change of ZP (**b**) versus the global strain measure $\delta$

### Inverse analysis and predictive simulations

Apart from the geometry and the reference solid volume fraction of the ZP, all other model parameters were calibrated by means of an inverse FE optimisation procedure. The measured force together with the volume change of ZP in the plate–plate compression in relaxation tests (experimental data in Fig. 6a, b, respectively) was used to compute two error functions, each of them defined by the weighted mean square error

16 $$\begin{aligned} e_q = \frac{\omega _q}{n \, \mathrm{{max}} [q^{\mathrm{{exp}}}] } \sum _{k=1}^{n}\left( q^{\mathrm{{exp}}}_k - q^{\mathrm{{model}}}_k \right) ^2, \end{aligned}$$

where $\omega _q$ and *n* represent the weight and the total number of sample points associated with the measured quantities *q*, respectively. Their sum was minimised in several optimisation steps, by means of the $\hbox {LiveLink}^{\mathrm{TM}}$ *for* $\hbox {Matlab}^{\textregistered }$ (version R2015b).

## Results

### Optical anisotropy in ZP

In the undeformed state, the slow axis orientation clearly shows the existence of two layers with highly aligned structures (Fig. 5b): an inner layer, approximately $50\%$ of ZP thickness with radial alignment, and an outer layer, extending roughly over the exterior $30\%$, with mainly tangentially oriented filaments. Moreover, while inner and outer layers are characterised by very low variation and almost constant orientation, the remaining middle region appears as a transition zone with enlarged variability (cf. normalised retardance plot in Fig. 5a).

### Mechanical response

#### Experimental results

The relaxation curves in plate–plate compression tests show a fast decrease of the force after the completion of the deformation ramp, whereas a continuous and slow force reduction is registered for longer time, without reaching a steady state during the sampled time interval (Fig. 6a). The detected volume changes are negligible for the OP but pronounced in the ZP, showing a peculiar trend inversion at the end of the loading ramp (cf. red arrow in Fig. 6b). Quantitatively, the measured volume overshoot $(v_{\mathrm{peak}} - v_{\mathrm{end}})/v_{\mathrm{end}}$ at the end of the ramp in terms of percentage of the long-term relative volume is given by $\xi ^{\mathrm {comp}}=(-4.03 \pm 4.23) \,\%$. At slow deformation rates, the response of the oocytes is nonlinear and significantly dissipative, as revealed by a wide closed hysteresis curve (Fig. 7a, b).

**Fig. 8:**
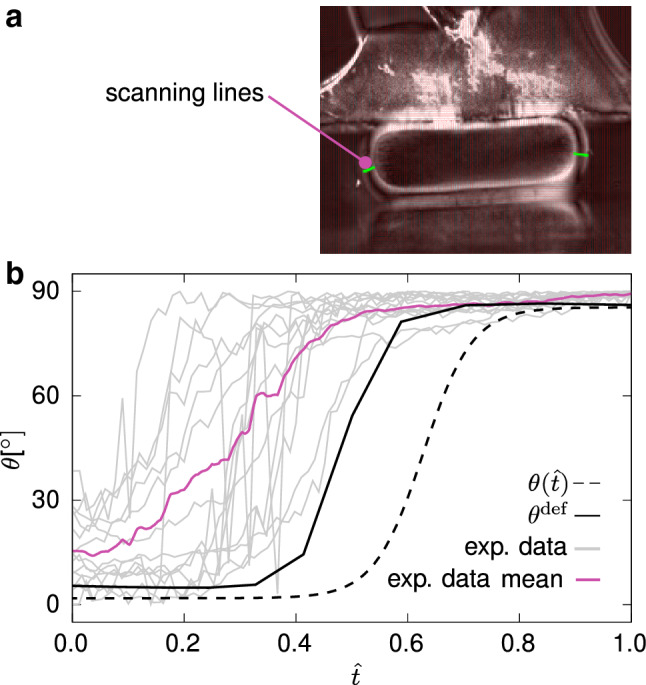
Example of orientation analysis obtained from the PolScope technique (**a**). **b** Experimental orientation curves (grey lines) and their mean (purple), and model prediction (black solid line). The initial fibre orientation along the normalised thickness ${\hat{t}}$ is plotted for comparison (black dashed line)

In Fig. 8, measurements of the orientation from different compressed oocytes are mapped onto the interval $[0,90]{^{\circ }}$ and plotted individually, due to the difficulty of defining uniquely the scanning line. A mean curve is also included (purple solid line) to give a qualitative representation of the trend. The scanning line is taken with a small offset from the direction perpendicular to the load (Fig. 8a). Figure 8b shows that, when compressed, as a result of the pressurisation of the OP, the internal zone of radially oriented proteins is reduced and this shift is correctly predicted by the model, from which the deformed orientation is calculated as $\theta ^{\mathrm {def}} = \mathrm {acos}\left( {1}/{N} \, \varSigma _{i=1}^N {({\varvec{m}}^i \cdot {\varvec{e}}_R)}/{\lambda _{\mathrm f}^i} \right)$, for the material line oriented in the direction specified by ${\varvec{e}}_R$.

**Fig. 9:**
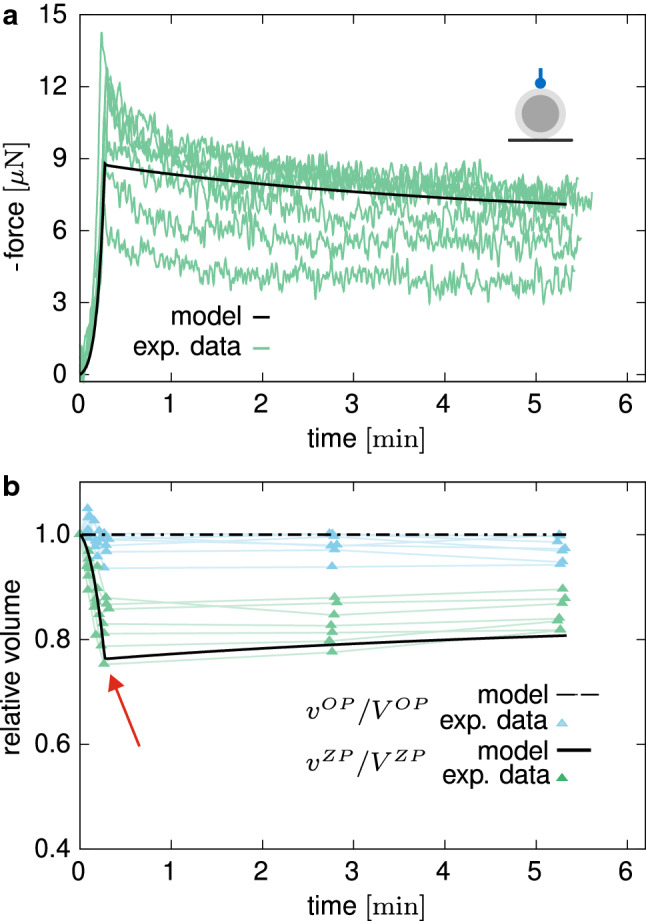
Indentation by means of a spherical indenter: measured force in relaxation experiment (**a**) and relative volume change (**b**) versus time

**Fig. 10:**
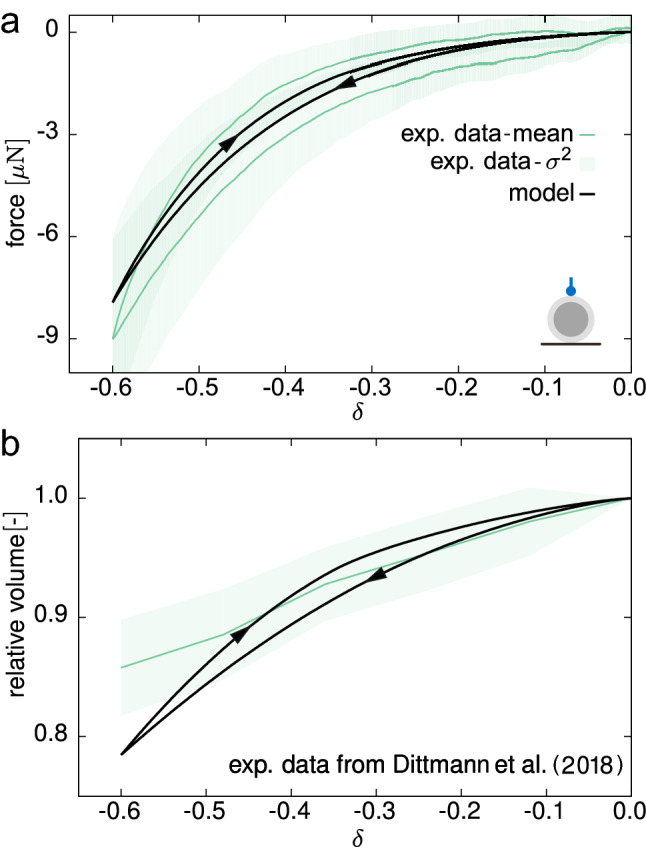
Indentation by means of a spherical indenter: measured force during the first cycle of a slow rate applied displacement (**a**) and relative volume change of ZP (**b**) versus global strain measure $\delta$

Compared to the compression load case, the indentation response is characterised by smaller values of both recorded forces and volume changes of the ZP (Fig. 9a, b). The deformation is heterogeneous and localised, and the volume recovery effect at the end of the load ramp, albeit present, is less notable than in the compression case (red arrow in Fig. 9b, $\xi ^{\mathrm {ind}}=(-2.83 \pm 3.11) \, \%$). Additionally, at the same maximum deformation in slow rate experiments, the dissipated energy in one cycle is less than the energy dissipation in the compression configuration (Fig. 10a, b).

#### Model calibration by relaxation compression tests

After parameter identification, the corresponding numerical results show sound agreement with the experimental data (Fig. 6a, b). When the *computational oocyte model* is rapidly compressed, the predicted time-dependent response results from both the fluid movement inside the ZP and the relaxation of the GPs. Distinctively, the latter is responsible for the characteristic inversion of the volume loss in the ZP (Fig. 6b). In fact, at fast rates, the stiffer fibres within the ZP reorient and pressurise the internal fluid. Therefore, its chemical potential increases, driving the fluid flux towards the external environment. As the time passes at unchanged deformed state, the fibres relax so that the pressure on the internal fluid and the chemical potential in the ZP reduce, driving the fluid back again.

#### Model validation

At first, the calibrated model was used to predict cyclic plate–plate compression tests at low deformation rates. While the simulations match the order of magnitude of forces (Fig. 7a), they underestimate the dissipated work in a cycle. Noteworthy and conversely, the relative volume change of the ZP in the loading part of the test is accurately predicted (Fig. 7b).

As a further validation of the calibrated model, the numerical results adequately predict the indentation response in both fast and slow deformation rates (Figs. 9a, b, 10a, b, respectively). The force trend and its order of magnitude are consistent with the experimental measurements (Figs. 9a, 10a), and the same features depicted in the case of compression are valid also in indentation, notwithstanding the more heterogeneous local deformation.

**Fig. 11:**
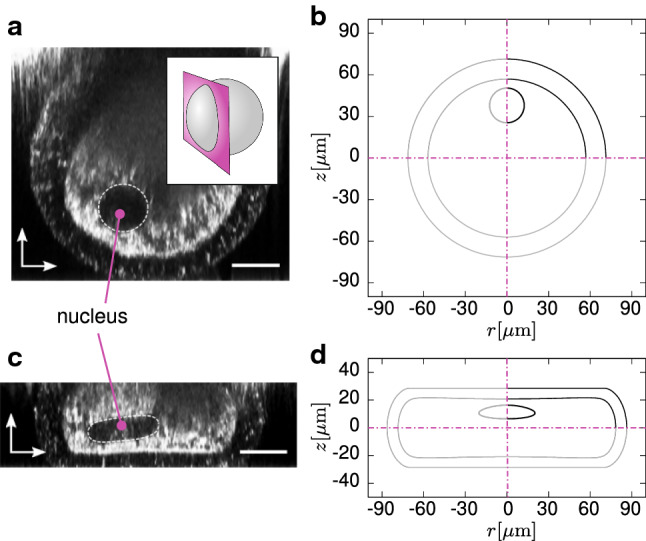
Undeformed (**a**) and deformed (**c**) nucleus in plate–plate compression at $\delta = 0.6$. The inset in **a** clearly shows that the image was sectioned at a plane not passing through the centre. Simulated undeformed nucleus (**c**) and corresponding prediction of its deformation (**d**). The undeformed radius of the nucleus in calculations is $r^{\mathrm{{nucleus}}}=12.5\,\upmu \hbox {m}$. The grey lines are plotted by symmetrising the profiles present in the first quadrant. Scale bars: $25\,\upmu \hbox {m}$

Finally, to get a coarse impression of how the nucleus would deform, we monitored the deformation of a circular sector placed near the compressed boundary of the oocyte (Fig. 11b, d), although typically the nucleus is not located in the median plane. The model captures the shear transmission across the OP, since the deformed shape of the sector is elongated. Nevertheless, we emphasise that we did not assign a specific strain energy to the nucleus.

## Discussion

### Volume changes in oocytes under compression

Image analysis was used to extract the volume changes for both ZP and OP with time. While the OP does not undergo any significant volume change, a peculiar time-dependent behaviour of the ZP is observed. The significant, time-varying volume decrease upon compression (Figs. 6b, 7b, 9b, 10b) and the inverted trend of the volume of the ZP at the beginning of the dwell phase (red arrows in Figs. 6b, 9b) suggest at least two concurrent dissipative mechanisms in the material. Considering the biphasic composition of the ZP—a porous solid network filled with a biofluid—a likely explanation of this time-varying mechanical behaviour can be ascribed to solid–liquid interactions and to viscoelasticity of the constituents. In fact, by means of our mathematical model, we rationalised the material compressibility of the ZP by the fast in/out flow of fluid through the external boundary, and we associated the long-term dissipative mechanism to inelastic weakening of the glycoprotein network under deformation, which is also responsible for the ZP volume recovery during the relaxation phase.

### Local orientations of the ZP

The radially and tangentially oriented structures in the inner and outer layers of the ZP, respectively, suggested by polarised microscopy, confirmed previous literature results regarding the orientation of proteins within the ZP (e.g. Keefe et al. 1997; Pelletier et al. 2004; Gu et al. 2010) and the retardance values from analogous techniques (Shen et al. 2005; Raju et al. 2007). In the middle zone, higher values of dispersion are inferred from the retardance data, and hence, it is typically assumed to be isotropic (see e.g. Keefe et al. 1997).

Notwithstanding, our experimental data yet show a smooth transition of the optical anisotropy along the thickness (Fig. 5a), with a small but nonzero polarisation also in the middle zone, suggesting that the orientation in this zone is not isotropic, i.e. not fully random. Under compression, the variable, non-ideal geometry of the oocytes complicated the definition of a predefined scanning direction. Notwithstanding, the orientation data show a common feature in that the amount of inner, radially distributed structures reduces compared to the undeformed state (Fig. 8b).

**Fig. 12:**
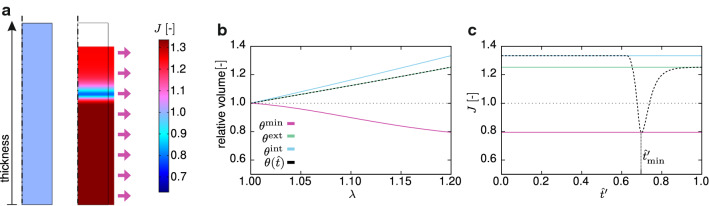
Equibiaxial test on a radially oriented strip of ZP (see blue rectangle in Fig. 4c). **a** Volume ratio *J* field in the reference and deformed configurations. Strip relative volume versus applied stretch $\lambda$ (**b**) and volume ratio *J* versus normalised current thickness ${\hat{t}}'$(**c**), for different orientation distributions

We note that we associated the aligned structures within the ZP with the GPs (cf. Goudet et al. 2008), whose tangential orientation in the outer layer is supported also by SEM imaging (Familiari et al. 2006). Conversely, such evidence is currently lacking for the expected radial GPs at the inside. Transzonal projections (TZPs), sent out from the surrounding cumulus cells, have been suggested as an alternative source of the observed optical anisotropy. Microscopy imaging could, in fact, explain their radial alignment within the inner layer (Coticchio et al. 2013; Guglielmo and Albertini 2013) and the tangential orientation at the outside (Guglielmo and Albertini 2013; Baena and Terasaki 2019). However, this hypothesis is not able to explain the angular distribution for the middle layer since TZPs are not known to change their direction inside the ZP. Ultimately, despite the uncertainty, the source of optical anisotropy is of little relevance with regard to the mechanical modelling performed, which is merely based on the assumption that optical and mechanical anisotropies agree. In particular, this assumption implies that the slow axis orientation characterises the alignment of those structural features that also act as a mechanical reinforcement, and thus generate the mechanical anisotropy.

### Time-dependent material behaviour

The presented outcomes have shown a distinct time-dependent behaviour of the oocytes. In particular, we emphasise two main features: (1) The existence of two separated timescales and (2) a notable dissipation also at slow deformation rates. Similar time-dependent characteristics were observed at tissue scale, e.g. for collagenous tissues (Mauri et al. 2015b; Bircher et al. 2016), but also in single-cell experiments on other cells. Particularly, poro-visco-hyperelastic models were adopted to rationalise AFM-based micro- or nanoindentation tests on chondrocytes (Nguyen et al. 2015; Florea et al. 2016), and two timescales were identified, associated with a short-term flow of intracellular liquid and a long-term viscoelastic response of the cytoskeleton (Florea et al. 2016).

Both fast compression and indentation experiments reveal relaxation of the measured force (Figs. 6, 9) and that no stationary state is reached within the analysed time interval (${\approx }300\hbox { s}$). Relaxation experiments on whole human oocytes also revealed long-term dissipative mechanisms (Liu et al. 2012; Shen et al. 2019). Defining the poroelastic and viscoelastic characteristic timescales as $\tau ^{\mathrm {pe}}\propto L^2/E/k^{\mathrm {ref}}$ (Grodzinsky 2011) and $\tau ^{\mathrm ve}\propto \nu /E$ (Gan and LAM 2008), respectively, where *E* is a representative elastic modulus, a relation between the two is obtained as $\tau ^{\mathrm {pe}}/\tau ^{\mathrm {ve}} \propto L^2/\nu /k^{\mathrm {ref}}$. Choosing *L* equal to the thickness of the ZP and using the parameter values specified in Table 1, one obtains $\tau ^{\mathrm {pe}}/\tau ^{\mathrm {ve}} \propto 10^{3}$. This indicates that the fluid flow through the pores of the ZP is significantly faster than the creep and relaxation of the ZP fibrous structures, and this coarse estimation suggests three orders of magnitude.

At slow-rate deformations, we observed a noticeable hysteresis in both configurations (Figs. 7, 10), a common feature with findings on different species (Kim 2013; Abadie et al. 2014; Shen et al. 2019). Previous studies on the isolated ZP (e.g. Nakamura and Hiramoto 1978; Kim 2013; Papi et al. 2013; Boccaccio et al. 2014) showed a marked dependency of ZP response on the deformation rate. In addition to this, OP viscosity was estimated to be almost one order of magnitude lower than ZP one (Shen et al. 2019), thus entailing a faster mechanical response. Together with these latter findings, our observations suggest that the ZP plays the major role in defining the time-dependent response. Correspondingly, the OP was modelled as a hyperelastic material in the present work, so that the time-dependent behaviour of the oocyte was exclusively associated with the visco- and poroelastic properties of the ZP. Among the two effects, the numerical results suggest that energy dissipation is provided mostly by the GP viscoelasticity, whereas the fluid flow within the ZP plays a minor role. Vice versa, in computational models of other cells, in particular chondrocytes, the relaxation behaviour under compression was related to the dissipation caused by recirculating intracellular fluid in a biphasic cytoplasm (Hou et al. 2018). These results cannot directly be compared or transferred to oocytes that are enclosed by a thick, compressible filamentous layer (ZP) whose volume is comparable to that of the ooplasm, and not by a thin membrane surrounding the cytoplasm. The governing mechanisms responsible for the time-dependent behaviour may therefore be considerably different.

### Equibiaxial deformation of a strip of ZP

In order to further comprehend the mechanical response of the ZP in terms of material behaviour, we investigated a simpler state of loading, by using the implemented model to study the hypothetical equibiaxial extension of a radial strip of ZP (Figs. 4c, 12a). We considered the elastic limit of a long-term response, for which $c_{\mathrm {fd}}=0$ since the fibres completely relaxed, and the fluid redistribution was completed. The results were obtained setting $\kappa ^\mathrm{ref, EB}= \kappa ^{\mathrm{ref}} \times 10^{10}$ and $1/\nu =0$. In Fig. 12b, the black dashed curve outlines the trend of the relative volume of the strip with increasing stretch, showing an overall increase in volume. The corresponding profile of the volume ratio at $\lambda =1.20$ (black dashed curves in Fig. 12c), plotted over the normalised current thickness ${\hat{t}}'$, is highly non-homogeneous, due to the anisotropy of the ZP and with a valley in the central region, displaying values lower than 1. In this zone, the material is densified and shows inverse poroelastic behaviour (see Ehret et al. 2017). To study the role of anisotropy, we also simulated the response with uniform orientation distributions (Fig. 12b, c). In particular, the purple line in Fig. 12b, c represents the relative volume trends when $\theta$ is kept constant along the thickness and equals the angle of the material elements with the largest volume loss, so that $\theta ({{\hat{t}}})\equiv \theta ^{\mathrm {min}}=62.29{^{\circ }}$. For comparison, the relative volume change and the *J*-profiles in the cases of homogeneous fibre orientations $\theta ({{\hat{t}}})\equiv \theta ^{\mathrm {int}}=\theta ({\hat{t}}=0)=1.88{^{\circ }}$ and $\theta ({{\hat{t}}})\equiv \theta ^{\mathrm {ext}}=\theta ({\hat{t}}=1)=85.34{^{\circ }}$, corresponding to the quasi-radial and quasi-tangential alignment at the internal and external boundary of the ZP, are plotted in Fig. 12b, c, respectively. From this hypothetical computational experiment, we conclude that the reorientation of the fibres in the middle zone is not sufficient to justify the large loss of volume registered in compression experiments (Fig. 6b). This can be instead facilitated by the pressurisation of the OP, which loads and compacts the fibres in the inner zone of the ZP (Fig. 2c).

### Limitations

Due to the multi-layer structure of oocytes and their large deformations during testing, the identification of the domain boundaries was non-trivial, in particular at the interfaces. After detection and determination of the boundaries of ZP and OP by an operator, volumes were approximated by summing the volumes of the tori obtained by rotating the respective square-shaped pixel area $a_{\mathrm{p}}$ about the vertical centre line. Moreover, the quantities obtained from the PolScope technique are essentially retrieved from 2D projections of a 3D network. Therefore, in the proximity of the inner and outer diameter of the ZP, where the alignment is substantially uniform, the PolScope analysis is very accurate. Conversely, the interpretation of the measured orientation angle in the middle layer is less accurate and affected by larger scatter. Nevertheless, the anisotropic distribution of the GPs in the model was prescribed considering the mean curve of the optical anisotropy angle, which is characterised by a smooth transition between the radial and tangential directions, in correspondence of the inner and outer layers of the ZP, respectively.

The hysteresis observed in cyclic tests (Figs. 7, 10) is not entirely captured by the current model, which points at additional phenomena to be considered in future developments. This concerns the already mentioned disregard of potential time-dependent behaviour of the OP, as well as damage and other inelastic effects which might take place at high compressive loads.

## Conclusions

In this study, novel data on the ZP microstructure were acquired by using the PolScope technique. Overall force-deformation curves in compression and indentation at slow- and fast-rate experiments were recorded together with the volume changes of both ZP and OP with time. This information revealed a previously unexplored time-dependent, compressible behaviour of the ZP, which was interpreted as the results of two separated dissipation mechanisms: the slow inelastic relaxation of ZP structures and the fast outflow of interstitial biofluid. The mathematical model served to rationalise the experimental observations and allows to investigate how mechanical loads affect the material at the micro-scale, in terms of fluid flow, levels of hydration, and fibre rearrangement, and could then provide useful insights with regard to the local state of the cell. Different from clinical and more applied research in this field, the present work does not aim at distinguishing between different states of oocytes through mechanical testing. Nevertheless, the results of this fundamental research point at several parameters that may serve this purpose, such as the two timescales of relaxation, the dissipated energy in a load cycle, and the volume change or change of optical anisotropy under compression. The performed modelling provides a connection between these parameters and structural characteristics of the cell, including the permeability of the ZP. The work underlines that mechanical indicators deduced from oocyte mechanical probing represent lumped metrics resulting from a range of physical and structural properties of the cell. The interpretation of these metrics in terms of cell properties requires either complementary experimental information, such as changes of shape and structure, or validated models.

**Table 1:** List of material parameters

*ZP*		
$\phi _{\mathrm s}^{\mathrm {ref}}$	0.1	(−)
*q*	0.49558	(−)
$c_0$	16.45	(kPa)
$c_1$	0.60281	(−)
$c_2$	0.26454	(−)
$c_3$	1.0523	(−)
$c_{\mathrm{f}}$	113.68	(−)
$c_{\mathrm{fd}}$	142.5	(−)
$\nu$	2.1978	(MPa s)
$k^{\mathrm{ref}}$	$6.6207\times 10^{-13}$	($\hbox {m}^{4}\, \hbox {N}^{-1}\, \hbox {s}^{-1}$)
$\alpha _{1}$	0.7361	(−)
$\alpha _{2}$	5.4603	(−)
$\beta$	−156	(−)
*OP*		
*c*	1	(Pa)
$\kappa$	1	(GPa)

## Data Availability

The data supporting the results of this study are contained in the article. Additional data are available upon reasonable request to the authors.
